# Pregnancy-Related Acute Kidney Injury: Experience of the Nephrology Unit at the University Hospital of Fez, Morocco

**DOI:** 10.5402/2013/109034

**Published:** 2012-12-20

**Authors:** Mohamed Arrayhani, Randa El Youbi, Tarik Sqalli

**Affiliations:** ^1^Nephrology Department, Hassan II University Hospital, Sidi Harazem Road, Fez 30000, Morocco; ^2^Epidemiology Department, Faculty of Medicine and Pharmacy, Sidi Harazem Road, Fez 30000, Morocco

## Abstract

*Introduction*. Acute kidney injury (PRAKI) continues to be common in developing countries. The aim of this paper is to study AKI characteristics in pregnancy and identify the factors related to the unfavorable evolution. *Methods*. This prospective study was conducted in the University Hospital Hassan II of Fez, Morocco, from February 01, 2011 to January 31, 2012. All patients presenting PRAKI were included. *Results*. 37 cases of PRAKI were listed. Their ages varied from 20 to 41 years old, with an average of 29.03 ± 6.3 years and an average parity of 1.83. High blood pressure was the most common symptom (55.6%). Thirty-nine percent were oliguric. PRAKI occurred during the 3rd trimester in 66.6% of the cases and 25% of the cases in the postpartum. Hemodialysis was necessary in 16.2% of cases. The main causes were preeclampsia, hemorrhagic shocks, and functional, respectively, in 66.6%, 25%, and 8.3% of the cases. The outcome was favorable, with a complete renal function recovery for 28 patients. Poor prognosis was related to two factors: age over 38 years and advanced stage of AKI according to RIFLE classification. *Conclusion*. Prevention of PRAKI requires an improvement of the sanitary infrastructures with the implementation of an obligatory prenatal consultation.

## 1. Introduction

Acute kidney injury represents a challenging clinical when it occurs during pregnancy. The worldwide incidence of pregnancy-related acute kidney injury (PRAKI) has decreased markedly in the past 50 years from 20–40% in 1960 to less than 10% in the current series through the legalization of abortion and improvement of antenatal and obstetric care [1].

In the recent years, the incidence of PRAKI has decreased in developed countries to only 1% to 2.8%. It is a rare complication of pregnancy following the disappearance of septic abortion and a better perinatal care [2, 3]. However, PRAKI is still frequent in developing countries; the incidence is around 4.2–15% [2]. Caring for women diagnosed with acute kidney injury is a real challenge for nephrologists and all the medical team.

PRAKI is usually caused by septic abortion in early pregnancy, by pregnancy toxemia, hemorrhages during pregnancy (antepartum and postpartum), and acute tubular necrosis in late pregnancy [4, 5]. Acute fatty liver is an uncommon cause of PRAKI. It occurs in the third trimester of pregnancy. Puerperal sepsis and thrombotic microangiopathy are seen in the postpartum period.

Acute tubular necrosis (ATN) is the most common condition with a good prognosis compared to other pathology like severe eclampsia, HELLP syndrome, and disseminated intravascular coagulation (DIC) where the glomerular involvement is preeminent [6, 7].

The aim of this study is to investigate the characteristics of PRAKI and determine the factors associated to unfavorable evolution of kidney injury.

## 2. Methods

This is an observational prospective study conducted in the nephrology department of Hassan II University Hospital (Fez, Morocco), between February 01, 2011 and January 31, 2012.

### 2.1. Criteria of Inclusion and Exclusion

All pregnant and postpartum patients who developed a PRAKI, with or without oliguria were induded. Patients with preexisting renal disease or renal insufficiency before pregnancy were excluded.

### 2.2. Data Collection

For this prospective study, data were collected from a questionnaire, validated by an expert committee belonging to the Scientific Committee of the Moroccan society of nephrology. We studied different aspects.

- Population: age, sex, history, and pregnancy care.
- AKI: clinical, biological, date of occurrence, and etiology.
- Changes in the final maternal renal function.
- The final maternal outcome.

### 2.3. Definitions

- Preeclampsia: eclampsia was defined by a set of three signs: hypertension (SBP ≥ 140 mmHg and/or DBP ≥ 90 mmHg), edema, and proteinuria after 20 weeks of gestation.
- Eclampsia was defined by the existence of generalized convulsions and/or loss of consciousness occurring during pregnancy or PP in preeclampsia.
- HELLP syndrome was defined by the existence of three main features: haemolysis, elevated liver enzymes, and low platelets count.
- Postpartum is the period beginning immediately after delivery and extending approximately three months.
- Acute kidney injury (AKI) was defined and classed according to RIFLE criteria based on changes in serum creatinine or changes in urine output, or both. The RIFLE (risk of renal dysfunction; injury to the kidney; failure of kidney function, loss of kidney function, and end-stage kidney disease) criteria include three levels of renal dysfunction and two clinical outcomes: “loss” and “end-stage renal disease” (ESRD).
- Evolution: unfavorable evolution means noncomplete renal recovery.

### 2.4. Statistics

Descriptive and univariate analyses have been conducted in collaboration with the epidemiology laboratory from Medicine and Pharmacy faculty of Fez, using the SPSS 11 software. Qualitative variables were expressed as percentages and quantitative variables as median or mean. The level of significance was set at less than 0.05.

## 3. Results

**Figure 1:**
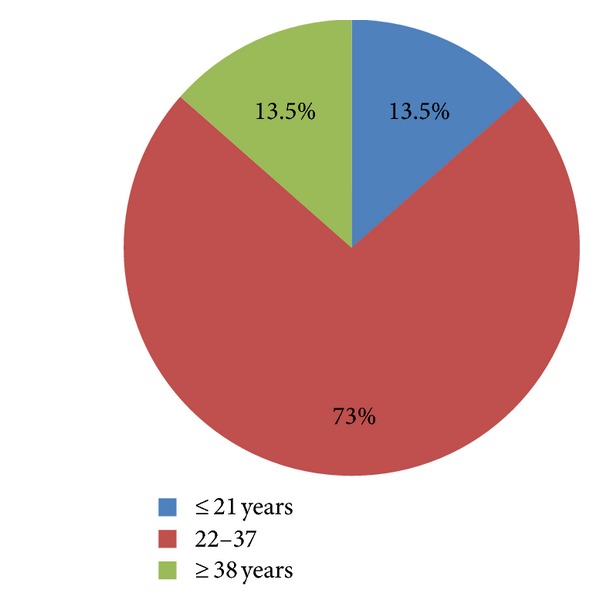
Age distribution in PRAKI patients.

**Figure 2:**
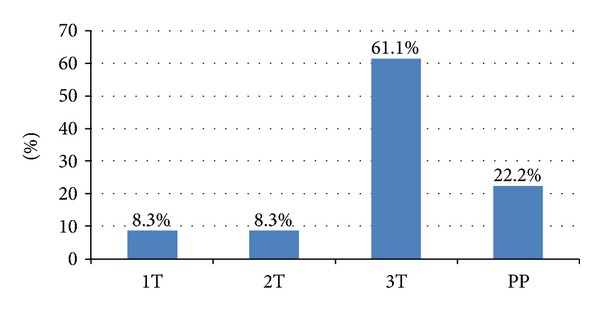
Gestational period at the discovery of ARF.

Total number of women with PRAKI was 37 in Hassan II University Hospital of Fez where 5600 deliveries have been performed in the study's time span. The incidence of PRAKI was near 0.66 percent. The patient's age ranged from 18 to 40 years with an average of 29.03 ± 6.3 years (Figure 1). The mean parity of the patients included in this study was 1.28 ± 1.13 (0 to 5). PRAKI occurred during the 3rd trimester in 61.1% of the cases, 22.2% in the postpartum period, and 16.6% in the 1st and the 2nd trimester (Figure 2). We noted a normal spontaneous vaginal delivery in 15 cases (40.5%), cesarean section in 15 cases (40.5%), and abortion in seven cases (18.9%). Hypertension was a common symptom present in 55.6%; thirty-nine percent were oliguric. Seizure occurred in 13.9%.

The stage of AKI according to RIFLE criteria was as follows: risk in 40%; injury in 27%; and failure in 33% of cases. The average creatinine was 34.8 ± 25.4 mg/L (8 to 105), proteinuria average 1.68 ± 1.53 (0–7 g/d) with nephrotic syndrome in 45.9% of cases, and nonnephrotic in 43.2%. Anemia was reported in 67.5% of patients, a thrombocytopenia in 56%, and hepatic cytolysis in 67% of patients.

Pregnancy toxemia is the most common cause of PRAKI (66.6%), followed by pregnancy hemorrhages (25%) and functional kidney injury (8.3%).

The evolution of our patients was marked by the use of vascular filling in 32.43%, vasoactive drugs in 5.4%, and transfusion in 42.9% of cases. Antihypertensive treatment by syringe pump was administered in 51.4% of cases and seven patients (18.9%) received diazepam. Five patients, 13.9%, presented a nosocomial infection. One of them died from respiratory infection.

Dialysis was required during hospitalization in 16.2%. Complete recovery of renal function was observed in 76% (28 patients) and partial recovery in 16.2% (6 patients). Two patients remained dependent on dialysis.

The univariate analysis assigned factors associated to unfavorable evolution of PRAKI which were older age, elevated creatinine level at admission, and RIFLE stage of acute kidney injury. The etiology of acute kidney injury was not associated to unfavorable evolution (Table 1).

## 4. Discussion

Acute kidney injury (AKI) occurring during pregnancy is a serious complication, involving the prognosis of the mother and the child [18]. Its specific physiopathology is strongly related to the physiological and hormonal changes occurring in pregnancy.

The PRAKI has become a rare complication of pregnancy in developed countries. For example, in France, the incidence of AKI in pregnancy has decreased from 40% in 1966 to 4.5% in 1978. This striking decline reflects the decrease of postabortion ARF and the better perinatal monitoring [19]. On the other hand, PRAKI is still common during pregnancy in developing countries, being responsible for a high maternal and fetal morbidity [20, 21].

The average age of onset for PRAKI is between 25 and 32 years according to various authors [22]. In our study, the average age was 29.03 ± 6.3 years, ranging from 18 to 40 years (Table 2). It appeared to be a factor significantly associated with unfavorable evolution (*P* = 0.01). In the literature, this factor was associated to increase perinatal complications, including premature delivery [8, 9, 22].

In our series, PRAKI was more frequent in the 3rd trimester (61%) and 22% in the postpartum. Similar results have been reported in Pakistan, where the majority of PRAKI occurred in the 3rd trimester (86%). Whereas in India, the PRAKI occurred during the postpartum in 75.6% of cases [10, 16].

The mean serum creatinine was 34.8 mg/L ± 25.4 with a maximum value of 105 mg/L and a minimum value of 14 mg/L. These results are comparable to those published by Randeree et al. [11], but significantly inferior to those found in the Pakistan series [16], Table 3.

In this study, the RIFLE stage in AKI is the related to unfavorable evolution (*P* < 0.02).

In most retrospective studies, preeclampsia eclampsia was reported to be a major cause of AKI during pregnancy. In our study, the main cause of AKI associated with pregnancy is PE (66.7%), with eclampsia in five cases (13.5%), and HELLP syndrome in 21 cases (58.3%). HELLP syndrome was described by Weinstein [23] in 1982, as a serious complication of severe PE, accompanied by a significant morbidity and high maternal and perinatal mortality.

The PE as a cause of AKI varies depending on the series from 12% in Pakistan [16] to 75.2% of cases in Turkey [17] (Table 4).

Septic abortions were the principal infectious cause of acute renal failure and a major public health problem in developing countries. However, there was no septic abortion in our series.

The obstetric hemorrhage was a significant cause of PRAKI. It was observed in 28% in Pakistan and 5% of cases in India [16].

Total recovery was obtained in 76% of the cases, which is similar to the results found in some other studies. Arora et al. [9], Goplani et al. [24], and Erdemoğlu et al. [15] reported a total recovery of renal function in 42%, 54.3%, and 61%, respectively.

Currently, maternal mortality due to PRAKI represents less than 10% in Europe and North America but remains high in the developing countries [25]. Recent studies in India have shown a maternal mortality rate around 20% [25]. In Turkey, this rate was 10.6% [15]. In Pakistan, Khalil et al. reported a maternal mortality rate of 15% in 2011 [8] compared to 33.3% of cases reported by Chaudhri et al. [26].

## 5. Conclusion

PRAKI remains a critical situation in developing countries where preeclampsia is the most frequent etiology, followed by sepsis and hemorrhagic shock. Advanced age and the RIFLE stage of AKI are associated with unfavorable evolution.

In this context, prevention is the best and least expensive solution. Preventing abortions, assuring a good perinatal care and a better management of obstetrical complications, are the crucial tools to implement this purpose.

## Figures and Tables

**Table 1:** Total recovery depending on the stage of the ARF.

Stage of ARF	Total recovery (%)	*P*
R	91.7	*P* < 0.02
I	41.7	
F	75.0	

**Table 2:** Age of onset of the PRAKI.

Studies	Age (years)	Extreme ages
Pakistan [8]	29	18–40
India [9]	25.8	15–35
Turkey [10]	31.6	17–46
Our study	29.03	18–40

**Table 3:** Comparison of creatinine in different series.

Author	Country	Period	Creatinine (mg/L)
Randeree et al. [11]	South Africa	1990–1992	47.7
Khalil et al. [8]	Pakistan	2006-2007	97
Altintepe et al. [10]	Turkey	1997–2001	57
Our study	Morocco	2011-2012	34.8

**Table 4:** Etiologies of PRAKI.

Authors	Countries	*n*	PE-E (%)	Sepsis (%)	DHD (%)	Hemorrhage (%)
Randeree et al. [11]	South Africa	42	48	29	—	—
Najar et al. [12]	India	569	15	50	7.5	5
Shaikhdr et al. [13]	Pakistan	294	25	11		28
Ventura et al. [14]	Uruguay	57	47	45.6	—	—
Erdemoğlu et al. [15]	Turkey	75	75.2	14.6		12
Ansari et al. [16]	Pakistan	116	12	31	—	—
Sivakumar et al. [17]	India	1353	30.5	47.4		18.5
Our study	Morocco	37	66.7	—	8.3	25

## Abbreviations

ATN: Acute tubular necrosis
AKI: Acute kidney injury
DIC: Disseminated intravascular coagulation
DHD: Dehydration
DBP: Diastolic blood pressure
SBP: Systolic blood pressure
PP: Postpartum
PR: Preeclampsia
PRAKI: Pregnancy-related acute kidney injury.
